# A Vision of State Medical Service

**Published:** 1918-11-09

**Authors:** G. T. K. Maurice

**Affiliations:** C.M.G.


### A VISION OF STATE MEDICAL SERVICE.

**By Colonel G. T. K. MAURICE, C.M.G.**

Unquestionably and for some years past, certainly ever since the failure of the Insurance Act to provide refreshing fruits and satisfactory conditions of medical attendance, the idea of a State Medical Service has been in the minds of men and has been discussed in desultory fashion. It may be interesting to consider a form the organisation might take.

It is necessary to have clear ideas of the objects to be aimed at in forming a State Medical Service. Let us say in sickness the best advice and treatment is to be placed within reach of every man, woman, and child of the community, and, perhaps rightly this matter should be placed first, thoroughly efficient measures to ensure the good health of the community are to be devised and properly carried out. The aims, then, are to be, by the guarding of the community against all the causes of ill-health, to keep it collectively and individually at a high level of good health, and by well-considered diagnosis and the best treatment known to science to restore to health such individuals as may become out of health.

Surgical and medical knowledge have stridden forward so rapidly in the last few decades that the means of diagnosis and treatment to the hands of the medical profession are far more elaborate, far more efficacious, and far more costly than they were heretofore. The guess-work of the old clinical diagnosis can be supplemented by $x$-rays, chemical tests, sera diagnoses, and so forth, and an accuracy, a certainty, attained that was formerly impossible. It is remarkable, and to the highest degree to the credit of the old clinical physicians, that so many of them by cultivating their powers of observation, and their power of judging from what their unaided senses saw, felt, and heard, should have been able to make so large a percentage of accurate diagnoses as they did. All of us make mistakes, and some more often than others, because everything in the end depends on fallible human judgment, and some are more fallible than others. But nowadays, given access to the apparatus necessary, the diagnosis can be confirmed or corrected in not one way only but many, by chemical tests, by microscope work, by exploratory incision, till almost it may be stated that, given reasonable time in a well-equipped institution, the most obscure case should be made plain before the last supreme proof of the *post-mortem* examination, which has so often shown us our mistakes.

But these elaborate processes are expensive: many require special buildings, costly apparatus, and men of long experience and special skill to apply them, and so, under present circumstances, they cannot always be brought to the service of the sick.

When little was known of the nature of disease the pill and the potion, natural force, and nursing, kindly enough but little skilled, were all that could be given beyond a few rough and ready surgical procedures. From that, sera and vaccines, new powerful drugs, anæsthetics, and aseptic surgery have brought about a wondrous change. But treatment by these means is an expensive matter, and entails the employment of highly skilled nurses. The useful, sympathetic, kindly woman, with little knowledge but much natural aptitude for the care of the sick, no longer suffices. So the wages of nursing have risen, and rightly, and should go up higher still.

The total result of all these changes is that the expense of making an accurate and fully confirmed diagnosis, and of giving thoroughly sound treatment and nursing, is so high that it is unattainable to most of the population except by charity. It could, however, be obtained by co-operation and mutual assurance, either by voluntary association or by submission to the taxation necessary to provision. Either is better than the pauperisation that comes of charity. The ideal is a healthy race, each man paying for himself or paying his share of the cost of maintaining the common health.

Sanitary science in all its wide ramifications has not yet come to its own. There is a beginning. In some large towns even a good deal is done to preserve health by preventing disease. But the important step of thoroughly educating the mass of the population has not yet been taken. All must learn that a healthy race implies parents without inheritable defects, strong and healthy in body and mind by gift of birth; implies restraint of the passions, avoidance of venereal disease, proper selection of foods, exercise in fresh air, avoidance of overcrowding, good ventilation of houses, healthy occupation, healthy amusements, and happy occupation of leisure instead of unwholesome pleasure. It is not reasonable for fathers and mothers with inheritable defects to expect healthy offspring; it is not reasonable for a man who works eight hours in a factory sitting over a machine to expect to maintain good health if his off-time is spent in the bar of a public-house, or in the stuffy, vitiated atmosphere of picture-palaces and music-halls. One of the first needs of health is to learn wisely to use leisure. More harm comes of misused leisure than from too long hours of work. All this must be taught. A State Medical Service should be an educating Service.

If, then, a State Medical Service were formed its functions should be executive and advisory. It should carry out measures to prevent disease, but the measures would emanate from legislative bodies. It should advise Parliament, county councils, corporations, and other public bodies. It should suggest legislation. It should treat the sick. It should educate the community in the ways of healthy living, and it should administer itself.

### A.M.S. and State Medical Service.

In considering the organisation of a new Service it is well to find a model to build after, to follow its lines, taking from it such as seems good, rejecting that not serviceable, and so shaping something suited to the end in view and capable of change, modification, and evolution.

A medical organisation that has functions such as have been stated for a State Medical Service is the Army Medical Service. This corps carries out measures to prevent disease, advises commanding officers and army committees, suggests regulations, treats the sick, and educates in the way of health by lectures, by pamphlets, by private talks the officers and men of the British Armies. Since its functions are so parallel to the functions suggested for a State Medical Service it may be that this organisation can be usefully considered as a model from which hints for the new organisation contemplated may be gained, though the differences of the conditions will necessitate wide divergences of the new from the tried.

**A Vision of State Medical Service**—*(continued)*.

The Army Medical Service with its head at the War Office in peace-time serves commands, districts of the Empire. A State Medical Service certainly will have its head in London, and equally certainly it will serve districts. Probably it would not be advisable for a district to be less than a county or a county borough. Large areas lessen the dangers and corrupt influences of local politics.

Each such district would necessarily have a chief medical man and a staff to help him. The head and staff, if the county be the unit, would be attached to the county council and have offices in the chief town of the county. This chief medical officer would be responsible for giving advice on all matters that concern the health of the county to the county council; he would inspect the sub-districts, the hospitals, the pathological institutes, the convalescent homes and so forth of the county, and report on them and the work done in them to the county council and to the head office in London, which would be in touch with Parliament. He would be the chief administrative medical officer of the county, and as such would be responsible for the proper and efficient employment of the members of the State Medical Service in his county.

In most counties it would be necessary to form sub-divisions, each under the control of a deputy chief medical man. To these men administrative work and inspection work would be deputed by the chief, and to that chief they would be responsible. The ends of the tentacles will be the medical men of the State Service working in the villages, the small towns, and districts of large towns.

In any typical English county there are small towns of from 3,000 to 10,000 inhabitants, separated from each other by agricultural areas in which are villages of various sizes, and hamlets and scattered cottages and farms. In the town will be three, four, five, or more medical men each running his own practice, or perhaps two or three in partnership, and often each desperately jealous of his *confrères* and fearful lest his patients may be filched from him. Such men attend the people in the town, perhaps some of them form the staff of a cottage hospital or hospital of slightly greater pretensions, and drive out to the villages round and about, attending a family here, a family there, and overlapping everywhere. One of them has the appointment of medical officer of health. It does not follow that he knows anything of sanitary matters beyond others, and frequently he will have to do violence to his conscience lest he offend some wealthy patient. Most will have some panel. Some will draw their income chiefly from that source. Others may assert themselves superior to that class of work, and, perhaps, find profit from the assertion. In some of the larger villages there will be men struggling to make a livelihood out of an agricultural panel, a few farmers, the village shopkeepers, and a poorly paid parish appointment. None of these men individually are in a position to avail themselves of modern methods of diagnosis, or of the latest and more costly means of treatment. Each is so busy running to and fro that he has not time to give to the processes, even had he the capital necessary to equip a laboratory and to fit himself out with microscopes, laryngoscopes, *x*-ray apparatus, and other special means of diagnosis, and a really good set of instruments. Jealousy or fear of giving advantage to his competitors prevents him from co-operating with them, and them with one another. This jealousy does not prevent friendliness nor good feeling. It entails a cautious attitude; but if fears materialise then there is resentment, open or suppressed. All make a living. Most strive to appear to do better than their books show. Seldom any is quite free from pecuniary anxieties. Such a condition of things is waste of force, of force that might be conserved and used for the betterment of the community, the more efficient treatment of the sick.

Other districts there are, mining and manufacturing districts of vast extent, where town runs into town and large grimy villages squat in dark valleys by stained brooks and rivers that run red, where the night sky glows with the glare of a thousand furnaces and the trees are sickly with chemical fumes. Here are large panel practices, often of several partners combined, and little society of a cultivated sort. The medical men live strenuously, with considerable pecuniary reward sometimes, but little of the charm and grace of life, and no time for experiment or study. Probably there is a well-equipped hospital somewhere in the district, in which a few men do interesting work, and into which the others get rid of their troublesome cases and see no more of them till the patients return cured and troublesome no longer.

There are large business towns, pleasant places often, social and with a varied society, with choice of life and many amenities. In them are hospitals, some excellent, some fair, and means of scientific diagnosis and treatment. But the hospitals have their staffs. The most of the medical men in the town have no standing in them at all. The hospitals serve as convenient receptacles for cases that worry and cannot pay; the staffs are available to advise on, and to operate on, those cases that worry and can pay. Quite commonly a medical man makes his living by treatment of the simple cases and advising consultations in the more difficult. He too often loses his professional enthusiasm, and his professional skill does not increase, under such uninspiring routine.

### New Arrangements under State Medical Service.

If there were a State Medical Service, to replace all this there might be some such arrangements as are to be detailed. In the small country town there would be established a senior and experienced medical man, and with him juniors of various degrees of seniority. This group of men would undertake all the medical work of a prescribed district. The sanitation of the town and the neighbouring villages would be under their supervision. All measures for the prevention of disease it would be for them to see properly carried out. All sick persons could demand their attendance. It would be for them to inspect schools and school children, and to carry out the educational instructions issued from headquarters.

The senior medical man would be concerned with the allotment of the work and with the general administration of his group, and be available for consultation. He would have an official residence near the hospital of the district.

The hospital and the group attached to it would vary with the importance and population of the district that they served. For a town of 10,000 inhabitants, with an agricultural district in a radius of six or seven miles containing, perhaps, twice as many more inhabitants, gathered in villages or scattered about it, say a hospital of 100 beds and an infectious hospital of thirty beds and a staff of twelve medical men; the senior medical man, the resident in the hospital, and ten men to get about and do the district work and to attend upon the patients in the hospital. The resident, as the word implies, would live in the hospital; the other men would live in such houses

**A Vision of State Medical Service—(*continued*).**

as suited them not far removed from it. Two or three would probably be detached for duty in such larger villages as the district contained, but they would never be out of touch with the hospital and the other members of their group.

The hospital would be the centre round which the work revolved and in which much of it was done.

Attached to it there would be the medical men's building, with common rooms and library and consulting rooms and offices. Here the medical men would assemble in the morning and meet the senior officer, and with him arrange the day's work. It might, of course, happen that one was out answering some call. He would have telephoned to the hospital that this was the case, and would have stated where he was and, also by the telephone, would keep the senior informed of how long he was likely to be kept and if he was available for other work in the part of the district to which he had been called.

As a matter of routine, to which the people of the district would easily become accustomed, during the earlier hours of the morning messages would have been coming in asking for medical attention, and on the visiting lists would be the names of those under treatment at their homes. Each ward of the town, each section of the district would have its own medical man in medical charge of it. The doctor would get the new names for his list each morning at the office and set off on his round. But before he set off he might have patients in the hospital to visit and prescribe for, and visitors at his consulting room. He would therefore go first to the wards he had to visit. If he found an operation necessary he would arrange with one or more of his brother State medical men to help him and to give the anæsthetic, or if he thought the case needed an operation beyond his experience he would call on that member of his group who was the most skilled as a surgeon to perform the operation for him, or consult with the senior on the advisability of telegraphing to the headquarters of the division or of the county for a special man to be sent down to do the operation. He would for any case have the advantage of the aid, assistance, and advice of any or all of his colleagues, and no fears about losing a patient to a competitor.

Let us imagine the hospital. In essentials it will not differ from the better hospitals of its size at present existing, but as it will serve every class of the population at need and as anyone will have a right to be treated in it, if hospital treatment be necessary; besides large wards that can be used free of charge on payment only of a subsistence charge there will be small wards of three or four beds for which a charge will be made, and small one-bed wards for which a considerably higher payment will be required. The nursing staff will be of the State Service, and in the wards, later on, when the Service is fully established and the inevitable State medical schools are in full swing, senior students may be doing the work of clerks and dressers. There will be a pathological laboratory, and in it every medical man will be enabled to carry out any tests or examinations that he wishes with the assistance and co-operation of that member of his group who is most skilled as a pathologist and of a trained laboratory attendant. The laboratory will not be equipped on a lavishly expensive scale. The more complicated and difficult examinations will be made at the chief county laboratory, situated in one of the largest towns of the county and attached to a large central hospital belonging to one of the divisions into which the county is divided.

### Treatment of Urban Areas.

HAVING sketched out slightly something of the central county organisation and something of the extreme end of the whole county organisation, it is convenient to rough in the intervening portion. It has been suggested that the county will be divided into sub-divisions under deputy-chief medical men. Since in most counties the county council offices are in the largest town of the county, and certainly in a large town, it is probable that this urban area would form one of the divisions. There would then be in it the chief medical officer of the county and his staff and a deputy-chief medical officer and his staff, and the staff that served the division. As this particular sub-division is by hypothesis urban and compact, it might not be necessary to divide it into sub-districts, a course that would be necessary in large areas of more scattered population, so one large central hospital would serve it. There would be a large medical officers' block, with more than one common room, an extensive library, a long range of consulting-rooms, dressing-rooms, nurses' waiting-rooms, offices, and so forth. Senior medical officer would be an important appointment. The man holding it would require a secretarial and clerical staff. The large hospital would have large wards for admission to which no charge would be made, and small wards use of which would entail a small payment, and single wards and small suites that would be rented at high figures to the wealthy sick. The chief laboratories and the research department of the county would be near by. The hospital, since it is in the chief town of the county, would serve more than its own division. To it would be sent material for elaborate examinations, and cases of special interest and difficulty from anywhere in the county. It might boast a medical school. It would be organised into medical and surgical divisions, each under a selected head, who would be consulted by the men treating cases in the wards and outside of them and who would be competent to give the best advice and the most highly skilled surgical aid. Any medical man of the sub-division would be entitled to send in any case he thought required treatment in hospital and to treat the case in the hospital himself, but subject to the supervision of the senior and specially selected head of the division. Or if he preferred, because of doubt of his own skill or because of pressure of other work, he would hand the case over to the head of the appropriate division for disposal. Special hospitals must, of course, be provided for infectious cases. In some towns there would be special hospitals for women and children, for lying-in, for eye cases, and so forth; in others such needs would be met by the setting aside of wards in the central general hospital.

The staff serving a populous district would be a large community. If one medical man to each 3,000 was the basis on which the strength of the general duty staff was calculated, the number so obtained, plus residents in the hospitals, pathologists, consultants, and so forth, is the strength of the district. And all these men would be colleagues, not competing but working together in comradely association, bound by the ties of a common service and of a great corps, to belong to which is of each the pride.

If the town were of very large size it would be divided into sub-districts of convenient size, each served by its own hospital. In such cases several variations of organisa-

### A. Vision of State Medical Service—(*continued*).

tion are possible. One county town might be a division divided into three, each with a hospital, a senior medical officer and staff. If so, it is probable that of these hospitals one would be larger, more fully equipped and staffed, and would receive by transfer cases that were of exceptional difficulty, or for one reason or another beyond the competence of the staffs of the smaller hospital that originally received them.

In very great cities each division would have its own fully equipped and fully staffed hospital, capable of dealing with any case that arose in the district, just as the great hospitals of London each serves a portion of the town and each is as efficient as another.

Other divisions of the county would centre round other important towns of the county. Obviously the number of the divisions would vary with the varying size and density of population of the counties. Some counties might be divided into three divisions only, others into six or seven, others into larger numbers still, and the division would be divided into sub-divisions of varying areas and varying populations, according to individual conditions and convenience for working. The division would always be large enough to support at least one hospital, the central general hospital of the division, which would be highly equipped for diagnosis, treatment, and research. The deputy-chief medical man of the division would have his office near it. To it cases would be sent from the smaller district hospitals, such as were first sketched, and attached to it would be well-equipped laboratories, staffed by highly skilled pathologists and chemists, with a due complement of subordinates, adequate for elaborate research, for the preparation and examination of specimens, for the preparation of sera and vaccines, and for the examinations necessary for reports on matters of public health.

It is not the intention to elaborate in every detail every possible organisation for a State Medical Service, but only to indicate a possibility and thus cause discussion and consideration of the question. Thus far the organisation suggested is a central head with offices and staff in London, county heads with offices and staffs in the chief towns of counties or county boroughs; county divisions of large size, each with a deputy-chief and his staff, and a central general hospital and appurtenances thereof; sub-districts of the divisions centred in some town of fair size and serving an area round it, an area in agricultural districts usually, with a radius of five to ten miles. In urban districts, convenient portions of a large town. Each district of a division with its hospital, its senior medical man, and his staff of such number of men as is necessary for the district. In the case most fully suggested about twelve was considered enough. It was a calculation based on a medical man to 3,000 of population. In other cases more or less would be the desirable number.

A variety of other details might be considered—such questions of administration as the passing of medical men temporarily from a reserve at the central hospitals, or from districts where the work was light, to divisions in which an epidemic was raging, the provision of substitutes for men sick or on leave, the selection of men for the jobs for which they were most well suited, and the hundred-and-one other questions that would arise and have to be tackled. But such details are beyond the necessities of a rough outline.

### How the Medical Profession may be Reconciled.

AN important question is, How will the medical men be reconciled to such a revolution?

The number of medical men and women in Great Britain is little, if any, greater than that necessary to get done all the work of a State Medical Service. The problem, then, is how can that number of medical men be induced to accept State service and State pay for their work in exchange for individualistic competitive practice and the earnings thereof?

If a man can see a pleasant life, interesting work, a reasonable reward for his work, a certainty in his old age, and a fair prospect for his wife and family if death should take him before the fulness of days comes to him, he will seldom hesitate to accept the career. It seems reasonable to expect in a State Medical Service a career that shall offer such conditions. If to them can be added honours and rewards to stimulate the able and ambitious, the Service would be attractive and would draw to itself a large proportion of the young men of natural talent. The variety of the work and the interest of it is greater than is offered by the work of many other branches of the Civil Service. If, therefore, the possible rewards are equal to those other branches of State work give, the Service will prove even more attractive than they. A difficulty that has to be faced in forming a State Medical Service is that none exists that can be built upon, and that a new Service would have to be made by enlisting in it the qualified medical men now earning their living as doctors. Those not successful and not capable of success would no doubt gladly come in, but how about the able and ambitious who are making money, who see a great career opening to them, who have great names and who are just the men wanted to organise, to administer, to treat the sick in the State hospitals, and to do the research work in the State laboratories? Many such may not see advantage to themselves, and many such will be hostile to the idea because they will dread that the establishment of a State Medical Service will interfere with their practices, cut down their rewards, and upset the plans of their lives.

There is no question but that such a profound change in the conditions of medical practice as the establishment of a thoroughly efficient Medical Service would entail could not be without disadvantage and even hardship to some. With this acknowledgment, and with recognition that revolutions may lead to appalling disasters before good results from them, consideration of the scheme may be continued.

(*To be continued.*)

